# Breakthroughs in Medicinal Chemistry: New Targets and Mechanisms, New Drugs, New Hopes–5

**DOI:** 10.3390/molecules24132415

**Published:** 2019-06-30

**Authors:** Arduino A. Mangoni, Jean Jacques Vanden Eynde, Josef Jampilek, Dimitra Hadjipavlou-Litina, Hong Liu, Jóhannes Reynisson, Maria Emília Sousa, Paula A. C. Gomes, Katalin Prokai-Tatrai, Tiziano Tuccinardi, Jean-Marc Sabatier, F. Javier Luque, Jarkko Rautio, Rafik Karaman, M. Helena Vasconcelos, Sandra Gemma, Stefania Galdiero, Christopher Hulme, Simona Collina, Michael Gütschow, George Kokotos, Carlo Siciliano, Raffaele Capasso, Luigi A. Agrofoglio, Rino Ragno, Diego Muñoz-Torrero

**Affiliations:** 1 Discipline of Clinical Pharmacology, College of Medicine and Public Health, Flinders University and Flinders Medical Centre, Bedford Park, SA 5042, Australia; 2 Formerly head of the Department of Organic Chemistry (FS), University of Mons-UMONS, 7000 Mons, Belgium; 3 Department of Analytical Chemistry, Faculty of Natural Sciences, Comenius University, Ilkovicova 6, 84215 Bratislava, Slovakia; 4 Regional Centre of Advanced Technologies and Materials, Faculty of Science, Palacky University, Slechtitelu 27, 78371 Olomouc, Czech Republic; 5 Department of Pharmaceutical Chemistry, School of Pharmacy, Faculty of Health Sciences, Aristotle University of Thessaloniki, 54124 Thessaloniki, Greece; 6 State Key Laboratory of Drug Research, Shanghai Institute of Materia Medica, Chinese Academy of Sciences, 555 Zu Chong Zhi Road, Shanghai 201203, China; 7 School of Pharmacy, Keele University, Hornbeam building, Staffordshire ST5 5BG, UK; 8 Laboratório de Química Orgânica e Farmacêutica, Departamento de Ciências, Químicas, Faculdade de Farmácia, Universidade do Porto, Rua Jorge Viterbo Ferreira 228, 4050-313 Porto, Portugal; 9 Interdisciplinar de Investigação Marinha e Ambiental (CIIMAR/CIMAR), Universidade do Porto, Terminal de Cruzeiros do Porto de Leixões, Avenida General Norton de Matos, S/N 4450-208 Matosinhos, Portugal; 10 LAQV-REQUIMTE, Departamento de Química e Bioquímica, Faculdade de Ciências da Universidade do Porto, Rua do Campo Alegre 687, 4169-007 Porto, Portugal; 11 Department of Pharmacology and Neuroscience, University of North Texas Health Science Center, 3500 Camp Bowie Blvd, Fort Worth, TX 76107, USA; 12 Department of Pharmacy, University of Pisa, Via Bonanno 6, 56126 Pisa, Italy; 13 Institute of NeuroPhysiopathology, UMR 7051, Faculté de Médecine Secteur Nord, 51, Boulevard Pierre Dramard - CS80011, 13344 Marseille CEDEX 15, France; 14 Department of Nutrition, Food Sciences and Gastronomy, Faculty of Pharmacy and Food Sciences, Institute of Biomedicine (IBUB) and Institute of Theoretical and Computational Chemistry (IQTC), University of Barcelona, Av. Prat de la Riba 171, E-08921 Santa Coloma de Gramenet, Spain; 15 School of Pharmacy, Faculty of Health Sciences, University of Eastern Finland, P.O. Box 1627, FI-70211 Kuopio, Finland; 16 Pharmaceutical & Medicinal Chemistry Department, Faculty of Pharmacy, Al-Quds University, POB 20002 Jerusalem, Palestine; 17 Department of Sciences, University of Basilicata, Viadell’Ateneo Lucano 10, 85100 Potenza, Italy; 18 i3S-Instituto de Investigação e Inovação em Saúde, Universidade do Porto, Rua Alfredo Allen 208, 4200-135 Porto, Portugal; 19 Cancer Drug Resistance Group-IPATIMUP-Institute of Molecular Pathology and Immunology of the University of Porto, Rua Júlio Amaral de Carvalho, 45, 4200-135 Porto, Portugal; 20 Department of Biological Sciences, FFUP-Faculty of Pharmacy, University of Porto, Rua de Jorge Viterbo Ferreira 228, 4050-313 Porto, Portugal; 21 Department of Biotechnology, chemistry and pharmacy, University of Siena via Aldo Moro 2, 53100 Siena, Italy; 22 Department of Pharmacy, University of Naples Federico II, Via Mezzocannone 16, 80134 Napoli, Italy; 23 Department of Pharmacology and Toxicology, and Department of Chemistry and Biochemistry, College of Pharmacy, The University of Arizona, Biological Sciences West Room 351, 1041 East Lowell Street, Tucson, AZ 85721, USA; 24 Department of Drug Sciences, Medicinal Chemistry and Pharmaceutical Technology Section, University of Pavia, Viale Taramelli 12, 27100 Pavia, Italy; 25 Pharmaceutical Institute, University of Bonn, An der Immenburg 4, 53115 Bonn, Germany; 26 Department of Chemistry, National and Kapodistrian University of Athens, Panepistimiopolis, 15771 Athens, Greece; 27 Department of Pharmacy, Health and Nutritional Sciences, University of Calabria, I-87036 Arcavacata di Rende, Italy; 28 Department of Agricultural Sciences, University of Naples Federico II, 80055 Portici, Italy; 29 ICOA, CNRS UMR 7311, Universite d’Orleans, Rue de Chartres, 45067 Orleans CEDEX 2, France; 30 Rome Center for Molecular Design, Department of Drug Chemistry and Technology, Sapienza University of Rome, P.le Aldo Moro 5, 00185 Rome, Italy; 31 Laboratory of Pharmaceutical Chemistry, Faculty of Pharmacy and Food Sciences, and Institute of Biomedicine (IBUB), University of Barcelona, Av. Joan XXIII, 27-31, E-08028 Barcelona, Spain

## 1. Introduction

Breakthroughs in Medicinal Chemistry: New Targets and Mechanisms, New Drugs, New Hopes is a series of Editorials which is published on a biannual basis by the Editorial Board of the Medicinal Chemistry section of the journal *Molecules*. In these Editorials, we highlight in brief reports (of about one hundred words) a number of recently published articles that describe crucial findings, such as the discovery of novel drug targets and mechanisms of action or novel classes of drugs, which may inspire future medicinal chemistry endeavors devoted to addressing prime unmet medical needs.

## 2. Inhibition of 5-Lipoxygenase-Activating Protein: A New Therapeutic Strategy to Combat Residual Inflammatory Risk in Patients with Ischaemic Heart Disease?

Highlighted by Arduino A. Mangoni

A significant number of patients suffer from coronary events despite being on maximal treatment with cardioprotective drugs. This suggests the presence of residual cardiovascular risk. Recent evidence highlights the key role of specific pro-inflammatory pathways in driving this residual risk and the need for novel, more effective treatments [1]. Pettersen et al. report the discovery and optimization of a series of novel inhibitors of 5-lipooxygenase-activating protein, which stimulates the production of leukotrienes, important mediators in the pathogenesis and progression of atherosclerosis [2]. In particular, compound AZD5718, (1*R*,2*R*)-2-{4-[3-methyl-1-(tetrahydro-2*H*-pyran-2-yl)-1*H*-pyrazol-5-yl]benzoyl}-*N*-(4-oxo-4,5,6,7-tetrahydropyrazolo-[1,5-*a*]pyrazin-3-yl)cyclohexanecarboxamide, demonstrated a favorable pharmacokinetic and safety profile in rats and dogs [3]. This led to phase-I studies in healthy subjects, which showed a strong relationship between plasma concentrations of AZD5718 and inhibition of both leukotriene B4, involved in inflammation, and leukotriene E4, a cysteinyl leukotriene also involved in inflammation [3]. Pending further investigations in patients with ischaemic heart disease, the results of this study suggest that inhibition of 5-lipooxygenase-activating protein might represent a promising therapeutic strategy to target residual inflammatory cardiovascular risk in specific patient populations.

## 3. Repurposing Ticagrelor: When an Antiplatelet Drug Could Emerge as a Potent Antibacterial Agent

Highlighted by Jean Jacques Vanden Eynde

**Figure 1:**
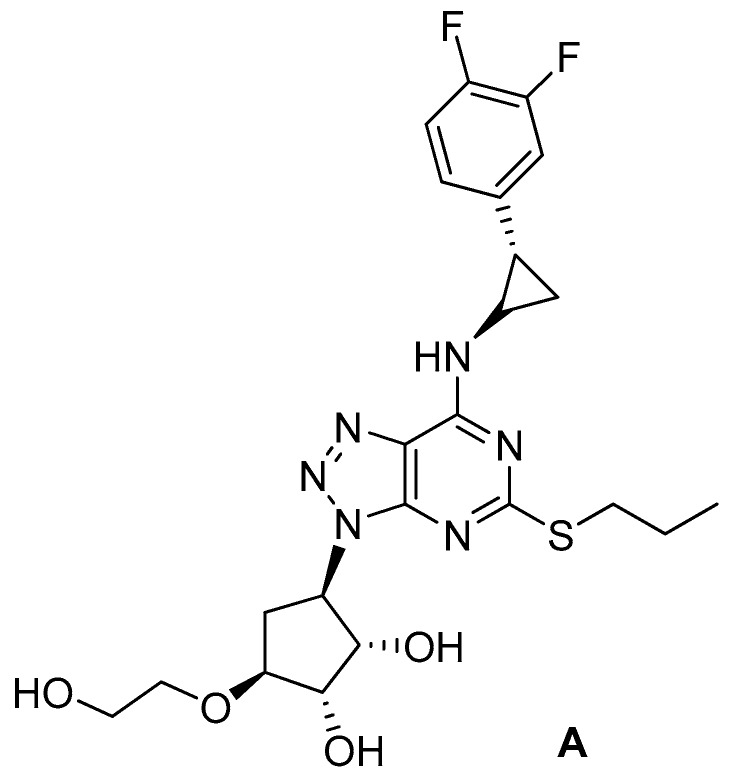
Chemical structure of ticagrelor.

Ticagrelor (**A**; CAS 274693-27-5) is a trisubstituted triazolo[4,5-*d*]pyrimidine acting as a blood-thinning agent by reversibly inhibiting the interactions between ADP and the P2Y12 protein (Figure 1). By preventing platelets aggregation, it reduces the risk of cardiovascular events in patients with acute coronary syndromes. The drug was approved in European Union at the end of 2010 and by the Food and Drug Administration in the middle of 2011. Recently, it was reported [4] that the drug could improve lung function in patients suffering from pneumonia. Inspired by those observations, P. Lancellotti et al. [5] screened ticagrelor and some of its metabolites for their activity, in vitro and in vivo (mouse models), against several strains of Gram-positive and Gram-negative bacteria. Interestingly, ticagrelor and one metabolite exhibited a bactericidal activity superior to that of vancomycin against selected antibiotic-resistant Gram-positive bacteria.

## 4. Anticancer Effective Tantalum Organo-Complexes

Highlighted by Josef Jampilek

Despite the approval of many anti-proliferative/anticancer drugs, cancer remains the second leading cause of death worldwide. Platinum-based alkylating antitumor chemotherapeutics, of which cisplatin is the best known, exhibit many adverse effects and, in addition, are associated with frequent tumor cell resistance. To innovate this class of drugs, a wide range of metal-based complexes with ruthenium, rhodium, osmium, iridium, molybdenum, iron, gold, and copper has been designed [6]. Recently, unique new electroneutral half-sandwich tantalum(V) dichlorido complexes containing pentamethylcyclopentadienyl (Cp*) and the double-deprotonated version of the Schiff base 2-{(*E*)-[(2-hydroxyphenyl)imino]methyl}phenol or 2-ethoxy-6-{(*E*)-[(2-hydroxy- phenyl)imino]methyl}phenol as ligands were reported as potential anticancer agents. In general, both tantalum organo-complexes [Ta(η^5^-Cp*)(*L*)Cl_2_] showed better cytotoxic potency in cisplatin-resistant cell lines (A2780R and HOS) as well as cisplatin-sensitive A2780 cells than cisplatin [7,8]. In addition, it is extremely important that they demonstrated markedly lower toxicity in non-tumor MRC-5 cells (IC_50_ > 50 µM) and in primary human hepatocytes (IC_50_ > 100 µM) than cisplatin and oxaliplatin [7,8,9]. Thus, these tantalum(V)-based complexes seem to be promising new anticancer agents.

## 5. Design, Synthesis, and Biological Activities of New Pyrazole Derivatives Possessing Both Coxib and Combretastatins Pharmacophores

Highlighted by Dimitra Hadjipavlou-Litina

In the past decade, studies have reported a growing body of data on different pyrazole derivatives and their innumerable physiological and diverse pharmacological activities.

Molecular hybridization is an approach in rational drug design where new chemical entities are obtained by combining two or more pharmacophoric moieties into a single molecule [10]. It is believed that the hybrid will present better bioactivity and pharmacokinetic profile than the original molecules. Combretastatin A-4 is a tubulin-binding chemotherapy drug that is structurally related to colchicine. Thi et al. [11] tried to discover novel multi-target agents with better anti-tumor activities than celecoxib. Twenty-one new aryl-substituted pyrazole derivatives possessing a *cis*-diphenylethylene scaffold were mostly synthesized by a one-pot approach to ethyl 1,4,5-triaryl-1*H*-pyrazole-3-carboxylates via an improved Claisen condensation–Knorr reaction sequence. The hybrids were tested against the three human cancer cell lines HT-29, Hep-G2, and MCF-7. The inhibition of NO production was studied. The results showed that incorporation of the two important pharmacophoric groups of the two original molecules celecoxib and combretastatin A-4 in a single hybrid molecule leads to better biological activities of the new coxib-hybrided compounds.

## 6. Novel Epoxyketone-Based Proteasome Inhibitors with Enhanced Metabolic Stability and Activity against PI-Resistant Models

Highlighted by Hong Liu

Proteasome inhibitors (PIs) have significantly improved the overall survival and quality of life of multiple myeloma (MM) patients. However, cancer resistance, either de novo or acquired, remains a major obstacle in expanding the clinical utility of PI drugs. Kyung Bo Kim and his co-workers have developed a series of peptide epoxyketones bearing a P1′-targeting moiety, of which the most potent Cfz−OH (IC_50_ = 29.4 nM) showed an IC_50_ value against H23 cells similar to that of carfilzomib (Cfz, IC_50_ = 18.3 nM) [12]. Moreover, Cfz−OH demonstrated improved potency by ~10-fold and 3-fold relative to Cfz in models of de novo and acquired Cfz resistance, respectively. On the other hand, the addition of a hydroxyl group adjacent to the epoxide ring of Cfz hindered the hydrolysis of the epoxide ring by inhibiting access to the active site of mEH. This work may provide us with a new direction for the development of new-generation PIs with enhanced metabolic stability and activity against PI-resistant patients.

## 7. Tyrosyl-DNA Phosphodiesterase 1 as a Novel Target to Enhance the Efficacy and Utility of Topotecan and Irinotecan

Highlighted by Jóhannes Reynisson

Many of the anticancer drugs used in the clinic are based on targeting the rapidly dividing malignant cells. These include DNA-damaging agents, which push the cancer cells into, e.g., apoptosis or necrosis. Topotecan and irinotecan are well-established topoisomerase 1 (Top1) poisons used against many cancer types. It has been shown that Tyrosyl-DNA phosphodiesterase 1 (TDP1) counteracts the effect of these drugs by unravelling the stalled DNA-Top1 complex, allowing cancer cells to survive the chemotherapeutic regime. Now reports have emerged of potent and selective small molecular inhibitors of TDP1 [13], which have the potential to dramatically increase the efficacy of topotecan and irinotecan as well as extend their application to other cancer types such as triple-negative breast cancer. In particular, it has been found that the adjunctive administration of TDP1 inhibitor with topotecan substantially reduces metastasis growth in Lewis lung carcinoma models, making TDP1 a very promising therapeutic target [13].

## 8. “KISS” Antimicrobial Peptides: “Keep It Simple and Stable” to Reach Selective New Antibiotics

Highlighted by Maria Emília Sousa

One breakthrough in the field of antibiotics has been the discovery of new antimicrobial peptides (AMPs). Membrane-active AMPs are appealing due to their low propensity for inducing the development of resistance but still present drawbacks such as toxicity, susceptibility to proteases, and manufacturing costs. Inspired by Svendsen and co-workers [14], who defined the pharmacophore of AMPs as two cationic charges and two bulky hydrophobic aromatic units, Boullet et al. [15], with the idea of keeping the AMPs simple and stable, synthetized new β^2,2^- and β^3,3^-bis-*homo*-ornithine/arginine peptides. This series of cationic peptides combined with the supertryptophan residue furnished AMPs with MIC values in the range of 2 to 16 µg/mL, active against both Gram-positive and Gram-negative bacteria. Structure-activity relationship studies were stablished, and a hit dipeptide emerged. This β^2,2^-amino acid derivative, easier to synthesize, showed to destabilize the bacterial membrane, revealed low hemolytic and cytolytic activities in mammalian cells, and was stable in human serum. The in vivo results highlight the potential of this new AMP for the treatment of sepsis in mice and the success of simple ideas for complex problems.

## 9. Amino Acid-Based Prodrugs of a Fosmidomycin Surrogate as Antimalarial and Antitubercular Agents

Highlighted by Paula A. C. Gomes

Malaria, tuberculosis (TB), and AIDS form the so-called “Big Three” triad of the most worrisome infectious diseases of the world. This terrible triad imposes a heavy toll especially to people from low- and middle-income countries (LMIC), where treatment options that are affordable to patients remain scarce. This is further aggravated when the etiological agents of the “Big Three” simultaneously affect the same person, causing AIDS/TB, malaria/AIDS, or malaria/TB co-infections, with quite discouraging prognoses. Medicinal chemistry efforts to find new low-cost strategies against the “Big Three” are not merely significant, but unquestionably urgent. A new opportunity towards this end was now opened upon the disclosure of fosmidomycin-inspired prodrugs as antimalarial and antitubercular leads, by Courtens et al. [16]. Fosmidomycin is a natural antibiotic whose antimalarial potential was unveiled at the turn of the 20th to the 21st century [17], paving the way towards the use of fosmidomycin in antimalarial combination therapies [18]. The recent work by Courtens et al. contributes to expand the chemical space around fosmidomycin and brings hope for future candidate drugs to tackle malaria/TB co-infections.

## 10. Horizon for a Prenatal Prevention of Cystic Fibrosis?

Highlighted by Katalin Prokai-Tatrai

Cystic fibrosis (CF) is a devastating genetic disease caused by mutations in the CF transmembrane conductance regulator (CFTR) gene that encodes the anion channel CFTR protein. The hallmark of CF is the accumulation of thick, sticky mucus that can damage multiple organs in the body, ultimately leading to diminished quality of life and early death. With the use of small-molecule CFTR potentiators, it may be possible to correct the defective CFTR channel in patients with specific CFTR mutations. Ivacaftor [*N*-(2,4-di-*tert*-butyl-5-hydroxyphenyl)-4-oxo-1,4-dihydroquinoline-3-carboxamide] is such a potentiator that can be given orally to patients as young as two years old. As symptoms of the disease may start before birth, Sun et al. [19] have used a ferret model of CF with specific mutations to pre- and postnatally administer ivacaftor. The authors found that prenatal administration to pregnant ferrets protected the fetuses against in utero development of CFTR defects and partially protected against the development of CF-related organ pathologies. At the same time, the authors also showed that postnatal ivacaftor withdrawal restored CFTR pathologies. These findings raise the hope that the prevention of CF-related pathologies may be possible before or soon after birth with timely administration of targeted therapies.

## 11. Small-Molecule PROTACs: A Promising Approach for the Development of Selective Drugs

Highlighted by Tiziano Tuccinardi

The PROteolysis-TArgeting Chimera (PROTAC) technology has emerged as a promising therapeutic tool that induces protein degradation by exploiting the ubiquitin proteasome system. This approach takes advantage of a two-headed molecule including a small molecule binder of a target protein (termed warhead), an E3 ligase-recruiting moiety, and a linker which connects these two portions of the molecule. By using the same warhead and the same recruited E3 ligase while modifying only the linker length and its attaching point on the E3 ligase-recruiting moiety, Crews and co-workers were able to identify two PROTACs that targeted individual isoforms of the p38 MAPK family [20]. Their results open a new way to develop selective drugs and highlight that the formation of the ternary complex (target protein–PROTAC–E3 ubiquitin ligase) is necessary but not sufficient for the PROTAC-induced substrate ubiquitination.

## 12. Screen Using Huge Virtual Docking Library to Accelerate the Discovery of Candidate Drugs

Highlighted by Jean-Marc Sabatier

In order to accelerate the discovery of candidate drugs, Lyu and collaborators [21] examined the virtual docking of 170 million molecules (from 130 known chemical reactions) against AmpC β-lactamase (AmpC) and the D_4_ dopamine receptor. According to this computer-based virtual screening, the authors selected 44 (for AmpC) and 549 (for the D_4_ dopamine receptor) candidate ligands/drugs which were chemically produced and experimentally tested for binding to their molecular targets. Interestingly, this approach allowed, in both cases, to discover the best-in-class molecules, including a novel family of highly potent (non-covalent) phenolate-based AmpC inhibitors. From the data, it therefore appears that bigger, ultra-large docking libraries are actually appropriate for the rapid and efficient discovery of new candidate drugs in the various areas of biomedical research.

## 13. Expanding the Range of Targeted Residues for the Design of Covalent Inhibitors

Highlighted by F. Javier Luque

The incorporation of a reactive “warhead” into a reversible recognition element underlies the design of chemical probes and drug candidates able to covalently bind to a target protein. Targeted covalent inhibitors (TCIs) combine improved potency and prolonged duration of action, but these advantages may be compromised by overly reactivity, which in turn may affect target selectivity. With the aim to expand the range of applicability of TCIs, Liu and coworkers have explored the possibility of designing lysine-target covalent inhibitors. In their work, they resorted to the usage of constant-pH molecular dynamics simulations to identify reactive catalytic lysines in a series of human kinases. These calculations disclosed the presence of reactive catalytic lysines (p*K*_a_ < 7.8), suggesting that they may occur in a rarely populated conformational state. Overall, this computational strategy gives rise to the design of suitably modified type II inhibitors to expand the arsenal tool of selective drugs targeting the human kinome [22].

## 14. Amino Acid Versus Phosphate Ester Prodrugs for Improving the Oral Bioavailability of Atazanavir

Highlighted by Jarkko Rautio

Atazanavir is an HIV-1 protease inhibitor suffering from suboptimal physicochemical properties that contribute to its poor absorption and susceptibility to both efflux and CYP-mediated metabolism. Both amino acid and phosphate ester prodrugs were produced to address these limitations. Although the direct phosphorylation of the secondary alcohol moiety resulted in the chemically stable phosphate monoester with significantly improved solubility, the prodrug failed to improve the oral absorption of atazanavir in rats due to inefficient bioconversion to the parent drug in the intestine. The phosphonooxymethyl prodrug with comparable solubility was more susceptible to intestinal bioconversion and provided a reasonable systemic exposure to atazanavir. Screening various amino acid prodrugs to overcome the barriers of both efflux and metabolism in addition to solubility-related issues resulted in the discovery of the L–Phe–Sar dipeptide prodrug that takes advantage of a self-immolative process of drug release. The prodrug provided a four-fold improved AUC and an eight-fold improved C*_24h_* value compared with an equivalent dose of the parent drug itself. This prodrug design appears to offer a viable strategy for addressing solubility issues of sterically hindered alcohols [23].

## 15. A General Method to Quantify Ligand-Driven Oligomerization from Fluorescence-Based Images

Highlighted by Rafik Karaman

To produce a therapeutic effect, a drug must first bind to specific receptors in the cell membranes. Receptors tend to change their molecular structure in a variety of ways during binding; only the right structure will “unlock” the drug’s therapeutic effect. Recently, a new method was developed by two research teams, using fluorescence-based images for assessing the therapeutic effects of drugs by matching them to their unique receptors [24]. This method has a great potential to enhance drug development and reduce the number of failing drug trials.

The method monitors the oligomerization process that occurs when a receptor generally exists as a single subunit but shifts to a multi-structure (an oligomer) in the presence of the drug, or vice versa.

The method was tested using fused fluorescent proteins and was validated on a receptor for the epidermal growth factor (EGF), whose malfunction is often linked to cancer. The activation of the receptor resulted in the generation of larger oligomers, as anticipated. The researchers then successfully applied the new method to a member of the G protein-coupled receptor (GPCR) family.

## 16. Reactivation of PTEN Tumor Suppressor for Cancer Treatment Through Inhibition of a MYC–WWP1 Inhibitory Pathway

Highlighted by M. Helena Vasconcelos

Reactivation of the phosphatase and tensin homologue deleted on chromosome 10 (PTEN), a tumor suppressor which is often inactivated in various human cancers, could be an effective approach for cancer treatment [25]. A recent publication by Lee et al. [26] identified a new potential target whose inhibition could restore normal PTEN functions: a ubiquitin E3 ligase (WWP1), which is an upstream regulator of PTEN dimerization and membrane localization, may be transcriptionally activated by the MYC proto-oncogene, and has previously been found overexpressed in some human cancers. In addition, through structure simulation and biochemical analyses, this study identified indole-3-carbinol (a natural compound found in cruciferous vegetables) as a potent pharmacological WWP1 inhibitor which reduced tumor growth in a mouse model of prostate cancer. This work may also encourage the discovery of new PTEN reactivators to treat cancer.

## 17. Derivatives of the Natural Alkaloid Matrine: New Anti-Fibrotic Tools in the Fight Against Idiopathic Pulmonary Fibrosis

Highlighted by Sandra Gemma

Idiopathic pulmonary fibrosis (IPF) is an interstitial lung disease that results in scarring and thickening of the lungs by largely unexplained mechanisms, with consequent progressive loss of lung function. Treatment of IPF relies on pirferidone and nintedanib, whose mode of action is not completely understood yet. Moreover, the efficacy of these drugs is unsatisfactory, so novel leads are urgently required. Li L. and colleagues [27] modified the structure of matrine, an alkaloid derived from a traditional Chinese medicine with known anti-fibrotic activity, by introducing specific substituents at the pyrrolizidine core. Most of the prepared derivatives showed improved anti-fibrotic activity compared to the reference alkaloid, coupled to reasonable selectivity indexes, and clear-cut structure–activity relationships were observed. Importantly, the authors dissected the biological pathway affected by the best anti-fibrotic compound arising from their study and discovered that it could involve the repression of TGFβ/Smad signaling by affecting the cytoplasm-to-nuclear translocation of Smad2/3. Taken together, the data presented in this study could contribute to the discovery of novel candidate drugs for the treatment of IPF.

## 18. Multi-Targeting Therapy for Glioblastoma: A Promising New Design

Highlighted by Stefania Galdiero

Brain cancer is a major public health problem worldwide and a leading cause of death. Glioblastoma is one of the most aggressive and common malignant brain tumors with a median survival of less than two years. Unfortunately, the success of glioma chemotherapy is hampered by poor drug penetration across the blood–brain barrier (BBB) and consequent low intratumoral drug concentration. Fan et al. effectively designed a multi-targeting hybrid carrier (Pep-MLHA hybrid nanoparticles (HNPs)) nanosystem based on a hyaluronic acid (HA)-modified polymer and a multi-targeting peptide. HNPs showed a strong penetration ability into the core of three-dimensional tumor spheroids and an efficient capability of crossing an in vitro BBB model. The authors also evaluated the in vivo brain tumor-penetrating capability and targeting properties of HNPs, as well as the therapeutic efficacy of docetaxel (DTX)-loaded HNPs. HNPs induced enhanced tumor localization, and DTX-loaded HNPs showed negligible systemic toxicity and enhanced therapeutic efficacy, with significantly improved survival rates of intracranial glioma-bearing rats. Such a design strategy is opening a promising avenue to develop multi-targeting hybrid systems as new delivery tools with superior therapeutic effects for glioma treatment [28].

## 19. New Potential Therapeutics for Gastrointestinal Stromal Tumors

Highlighted by Christopher Hulme

**Figure 2:**
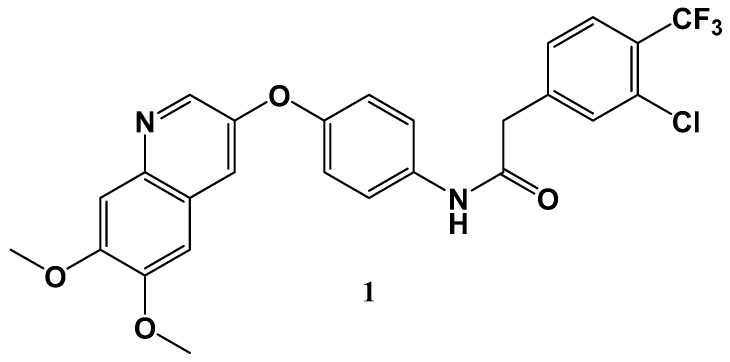
Structure of CHMFL-KIT-64.

Gastrointestinal stromal tumors (GISTs) represent the most prevalent mesenchymal tumors in the digestive tract where discovery of the c-kit mutant was a major breakthrough in GIST pathology, affording a drugable therapeutic target. Liu et al. [29] have recently discovered a Type 2 inhibitor 1 (CHMFL-KIT-64) (Figure 2) which is potent against both wild-type c-KIT and the array c-KIT T670I mutants. The molecule exhibits and excellent PK profile in several different species, demonstrating promising in vivo efficacy c-KIT mutant mediated mice models in addition to c-KIT wild-type primary cells, demonstrating imatinib resistance. As such, the overall profile of the molecule enticingly indicates that it may be a possible clinical candidate for gastrointestinal stromal tumors.

## 20. Directly Targeting Riboswitches with Small Molecules to Regulate Gene Expression: Identification of PreQ1 Riboswitches Ligands

Highlighted by Simona Collina

Riboswitches are regulatory, noncoding RNA aptamers that regulate gene expression by binding to specific small molecules. Particularly, the PreQ_1_ riboswitch governs the expression of genes responsible for the biosynthesis of queuosine (Q), a factor of key importance in bacterial virulence. Its cognate ligand is PreQ_1_ (7-aminomethyl-7-deazaguanine), a modified guanine-derived nucleobase [30].

In their study, Schneekloth and co-workers discovered a new class of small molecules that bind directly to PreQ_1_ riboswitches. A small-molecule microarray (SMM) screening on the aptamer domain of the PreQ_1_ riboswitch of *Bacillus subtilis* led to the identification of 20 hit compounds from a library of 26,227 molecules. Compounds showing selective binding over other RNAs were fully characterized through a series of orthogonal biophysical experiments, including NMR techniques, fluorescence titration, and in-line probing experiments. As a result, the authors identified three molecules as valuable starting material for developing compounds specifically tailored to target the bacteria-specific Q biosynthetic pathway.

This work highlights challenges in developing ligands for functional RNAs and in understanding the behavior of small synthetic compounds that bind to and modulate the function of complex RNAs.

## 21. Discovery of Protein–Protein Interaction Stabilizers by Site-Directed Fragment-Based Screening

Highlighted by Michael Gütschow

In contrast to disruptors of a protein–protein interaction (PPI), low-molecular-weight stabilizers of PPIs are relatively scarce [31]. The groups of Christian Ottmann at the Laboratory of Chemical Biology, Eindhoven University of Technology, and Michelle R. Arkin at the Department of Pharmaceutical Chemistry and Small Molecule Discovery Center, University of California, together with collaborators have provided a highly interesting study on a site-directed screening technique to successfully select fragments that enhance the affinity between protein partners [32]. Using the example of the interaction between the hub protein 14-3-3 and the phosphorylated peptide derived from Estrogen Receptor α (ERα), a 1600-member disulfide library was applied, capable of generating fragment conjugation through disulfide trapping. Conjugation was analyzed by intact protein MS and exemplarily studied in fluorescence anisotropy experiments. The authors identified stabilizers that increase the 14-3-3/ERα affinity up to 40-fold. The molecular mechanism of cooperativity was impressively elucidated by means of multiple X-ray co-structures.

## 22. The Short-Chain Fatty Acid Pentanoate as A Potent Immunomodulatory Molecule

Highlighted by George Kokotos

Short-chain fatty acids (SCFAs) such as acetate (C2), propionate (C3), and butyrate (C4), which are generated by bacterial fermentation of dietary fiber in the intestinal lumen [33], have attracted a lot of interest because they exert immunomodulatory effects. A recent study demonstrates that the physiologically abundant SCFA pentanoate (C5) is a potent regulator of immunometabolism [34]. It induces IL-10 production in lymphocytes by reprogramming their metabolic activity towards elevated glucose oxidation. In experimental mouse models, it mediates protection from autoimmune pathologies, shows a potent histone deacetylase-inhibitory activity in CD4^+^ T cells, inhibits the generation of small-intestinal Th17 cells, and ameliorates segmented filamentous bacteria (SFB)-promoted inflammation in the central nervous system. Thus, pentanoate might be of therapeutic relevance for the treatment of inflammatory and autoimmune diseases.

## 23. Small-Molecular-Weight Synthetic Benzophenone Derivatives as New Potential Breast and Prostate Anticancer Drugs

Highlighted by Carlo Siciliano

Brest and prostate cancers are two common invasive tumors causing death in women and men, respectively. The preferred treatment for prostate cancer is hormone therapy, but patients can develop resistance to long-time androgen deprivation therapy. Surgery, radiation, and/or chemotherapy are often drastic treatment against breast cancer. Novel drugs and targeted delivery of clinically approved anticancer drugs to tumor cells can ameliorate the outcome of these kinds of cancer, reducing toxicity and unwanted side effects. The effectiveness and benefits of synthetic small-molecular-weight compounds as potential anticancer drugs are currently under assessment. These compounds are often affordable by structural tuning of natural molecular frameworks. Recently, five differently substituted 2-hydroxybenzophenones were synthesized by a general scheme in which the 1,4-conjugate addition/intramolecular cycloaddition/dehydration of nitromethane was a key step in the tuning of natural chromone scaffolds [35]. All new benzophenones were tested in vitro for their antiproliferative activities on different breast and prostate cancer cell lines. A detailed investigation highlighted very good cytotoxicity effects, resulting in cell detachment/death, and high specificity for all derivatives with respect to doxorubicin used as a reference.

## 24. Sex-Specific Leukotriene Formation is Causative for Sex Dimorphism in Murine Asthma-Like Features

Highlighted by Raffaele Capasso

Asthma is a common airway inflammatory disease whose incidence and severity are age- and sex-dependent. In particular, this disease preponderates in women versus men. To date, sex has not been considered as a discriminant factor in the pharmacological therapeutic approach of asthma.

Leukotrienes (LT) are lipid mediators with a pro-inflammatory role in asthma, and recently a sex disparity in their production during allergen sensitization has been demonstrated [36]. Particularly, allergen sensitization selectively increased pulmonary LT biosynthesis in female mice. These sex differences in LT synthesis significantly affect the development of asthma-like features such as airway hyperreactivity and lung inflammation, which are more severe in females. In addition, according to evidence from other inflammatory conditions [37,38], different types of LT modifiers (i.e., montelukast and zileuton) currently used in therapy, prevent asthma-like features only in female mice. These data suggest a sex-dependent LT production as a basic mechanism of sex dimorphism in allergic asthma and strongly prompt for potential gender-tailored asthma therapy.

## 25. New Asymmetric Pd-Catalyzed Synthesis of Nucleoside Analogs

Highlighted by Luigi A. Agrofoglio

Nucleoside analogs are used not only as building blocks in the genetic code but also as biosynthetic intermediates, energy donors, metabolic regulators, and cofactors in enzymatic processes. Thanks to the broad spectrum of their biological functions, they are identified as pharmaceutical lead anticancer, antiviral and antibacterial compounds, for the treatment of metabolic and genetic diseases. They are mostly synthesized by the coupling of activated bases with anomerically activated sugars using traditional Vorbruggen glycosylation, with poor yields and diastereoselectivities with substrates lacking a 2′ assisting group. The group of Barry M. Trost at Stanford University [39] has developed a new catalyst system that enables the enantioselective synthesis of pyrimidine nucleoside analogs from acyclic *N*-heterocyclic amide ethers, with a broad substrate scope in excellent yields (up to 96%) and diastereo- (>20:1) and enantioselectivity (up to 99.5% ee). The new Pd catalyst system (Cp(allyl)Pd (cat.) and (S-S)-ligand (cat.) in presence of NIS in CH_2_Cl_2_, enables the enantioselective synthesis of pyrimidine nucleoside analogs bearing an iodide functional group handle for further functionalization, with a broad substrate scope via iodoetherification. 

It is believed that these data have a great potential to design pyrimidine nucleoside derivatives from 5- to 12- membered rings.

## 26. Experimental Data Shed Light on the Molecular Basis of Cancer Resistance

Highlighted by Rino Ragno

Venetoclax (Ven ABT-199) is the first BCL-2 antagonist approved for Chronic Lymphocytic Leukemia (CLL), and Acute Myeloid Leukemia (AML) therapy in 2016. Ven molecular mechanism of action displays a direct of protein-protein interfering with BH3 preventing BCL-2 anti-apoptotic effect. Birkinshaw et al. based on experimental structural data reported a detailed comparison between ABT-199 complexed either with BCL-2 or its mutated forms, being the G101V mutation mainly responsible for the CML acquired resistance [40]. The valine replacement induces a conformational change causing a rearrangement of venetoclax binding mode weakening the drug affinity by a negative modulation with sub-pockets P2 and P4. This was further assessed by inspection of G101A mutation which did not display any conformational rearrangement. F104L mutation structural analysis did not show any substantial conformational changes, but just a slight alteration of the Ven’s chlorophenyl moiety binding. Similar observations resulted also by inspection of S55746, a BCL-2 selective compound, currently under development [41]. In conclusion the paper clearly demonstrates the role of structural investigation to shed light on molecular mechanisms for the acquisition of cancer resistance.

## 27. PROTACing the UnPROTACable

Highlighted by Diego Muñoz-Torrero

**Figure 3:**
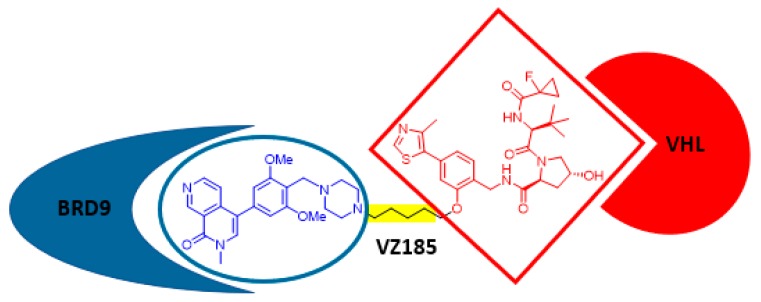
Chemical structure of VZ185.

PROteolysis TArgeting Chimeras (PROTACs) are hybrid compounds in which a ligand for an E3 ligase and a ligand for a target protein (protein of interest, POI) are connected through a linker. Upon formation of a ternary complex POI–PROTAC–E3 ligase, the POI is ubiquitinated and degraded by the proteasome. Thus, PROTACs have emerged as promising drug candidates and powerful tools for target validation. An important question that remained unclear was whether optimal POI–E3 ligase pairs exist or whether any potential combination could be addressed. The latter hypothesis has been soundly addressed by the group of Ciulli, with a novel PROTAC that targets a POI–E3 ligase pair considered unPROTACable: BRD9, an active bromodomain which is involved in MYC transcription and proliferation of leukemic cells and the von Hippel–Lindau (VHL) E3 ligase. After the elegant design of three generations of compounds, VZ185 (Figure 3) was identified as a potent, fast, and selective degrader of BRD9 (half-degrading concentration 1.8 nM; maximal degradation 95%), with very potent cytotoxicity on leukemia EOL-1 and malignant rhabdoid tumor A-204 cells. A plethora of POI–E3 ligase combinations are awaiting the discovery of novel PROTACs [42]!
